# Interplay of Nutrients, Temperature, and Competition of Native and Alien Cyanobacteria Species Growth and Cyanotoxin Production in Temperate Lakes

**DOI:** 10.3390/toxins13010023

**Published:** 2021-01-01

**Authors:** Ksenija Savadova-Ratkus, Hanna Mazur-Marzec, Jūratė Karosienė, Jūratė Kasperovičienė, Ričardas Paškauskas, Irma Vitonytė, Judita Koreivienė

**Affiliations:** 1 Department of Algology and Microbial Ecology, Nature Research Centre, Akademijos Str. 2, LT-08412 Vilnius, Lithuania; jurate.karosiene@gamtc.lt (J.K.); jurate.kasperoviciene@gamtc.lt (J.K.); ricardas.paskauskas@gamtc.lt (R.P.); irma.vitonyte@gmail.com (I.V.); 2 Division of Marine Biotechnology, Institute of Oceanography, University of Gdańsk, al. Marszałka Piłsudskiego 46, PL-81-378 Gdynia, Poland; hanna.mazur-marzec@ug.edu.pl; 3 Institute of Oceanology, Polish Academy of Sciences, Powstańców Warszawy 55, PL-81-712 Sopot, Poland

**Keywords:** *Aphanizomenon*, *Chrysosporum*, *Planktothrix*, *Sphaerospermopsis*, microcystins, saxitoxin, non-ribosomal peptides, bloom-forming cyanobacteria, Europe, freshwater shallow lakes

## Abstract

Global warming and eutrophication contribute to formation of HABs and distribution of alien cyanobacteria northward. The current study assessed how alien to Europe *Sphaerospermopsis aphanizomenoides* and *Chrysosporum bergii* will co-occur with dominant native *Planktothrix agardhii* and *Aphanizomenon gracile* species under changing conditions in temperate freshwaters. The experiments were carried out to examine the effect of nutrients and temperature on the growth rate of cyanobacteria, production of cyanotoxins, and interspecies competition. The highest growth rate was determined for *A. gracile* (0.43 day^−1^) and *S. aphanizomenoides* (0.40 day^−1^) strains at all the tested nutrient concentrations (IP and IN were significant factors). *S. aphanizomenoides* adapted to the wide range of nutrient concentrations and temperature due to high species ecological plasticity; however, *A. gracile* was able to suppress its dominance under changing conditions. Regularity between tested variables and STX concentration in *A. gracile* was not found, but IP concentration negatively correlated with the amount of dmMC-RR and other non-ribosomal peptides (NRPs) in *P. agardhii* strains. The relative concentration of NRPs in nontoxic *P. agardhii* strain was up to 3-fold higher than in MC-producing strain. Our study indicated that nutrients, temperature, and species had significant effects on interspecies competition. *A. gracile* had a negative effect on biomass of both alien species and *P. agardhii*.

## 1. Introduction

Global warming and anthropogenic eutrophication simultaneously contribute to formation of harmful algal blooms (HABs) worldwide, which are the biggest threat to freshwater ecosystems. Cyanobacteria may comprise over 70% of the total phytoplankton biomass in eutrophic lakes [1] at biomass reaching a high alert level (>12.5 mg L^−1^), according to the World Health Organization [2] recommendations for bathing waters. Toxicity of the cyanobacteria bloom depends on the structure of cyanobacterial community and the biomass of toxin-producing strains [3,4]. Among cyanotoxins, hepatotoxic microcystins (MCs) are the most common in European water bodies [5,6]; less frequently, the occurrence of neurotoxins and other bioactive metabolites of cyanobacteria has been reported from freshwaters of the continent. The results of the European Multi Lake Survey (EMLS) revealed MCs occurrence in 93%, while cylindrospermopsin (CYN) and anatoxin (ATX) were detected in 39% of the 137 EMLS lakes [6].

Anthropogenic eutrophication increases bioavailable nitrogen (N) and phosphorus (P) concentrations in freshwaters; however, it is still debated which of these two elements is more important for the HABs formation. Some authors refer to phosphorus as the main limiting element for primary production and magnitude of the bloom [7], while the others have shown that both P and N can control the blooms’ occurrence and intensity [8,9,10]. According to Klausmeier et al. [11], the cyanobacteria response to N:P is species-specific; therefore, the ratio might be important for species composition in the water body. Bloom-forming cyanobacteria (*Dolichospermum*, *Aphanizomenon,* and *Cylindrospermopsis*/*Raphidiopsis*) are able to fix N_2_ [12] and have strong competitive capabilities under nitrogen-limiting conditions [13,14]. However, Dolman et al. [15] revealed that various N_2_ fixing species have different preferences regarding N vs. P concentrations. Non-diazotrophic cyanobacteria such as *Microcystis* and *Planktothrix* dominate in the shallow eutrophic lakes under N-replete conditions [16,17] and evolved the capability to store the nutrients in the cells under limiting conditions [18,19]. Current understanding is insufficient to determine how concentrations of nutrients and their ratio challenge individual species. Therefore, it is crucial to assess how changing nutrients will result in ‘‘winners’’ and ‘‘losers’’ in phytoplankton assemblages [20].

In temperate lakes, competition for the main resources (e.g., nutrients, light) determine the complexes of dominant and co-occurring native cyanobacteria species [21,22]. However, due to climate change, the recent spread of alien cyanobacteria has put a great risk such that new competitive species may break the steady balance of native cyanobacteria dynamics and structure. The successful spread of *Chrysosporum bergii* and *Sphaerospermopsis aphanizomenoides* alien species in European temperate regions such as Poland [23,24], the Czech Republic [25], and Germany [26] in recent years has already been recognized. Lithuania is known as one of the most northern points for those cyanobacteria, and both species were detected in the shallow eutrophic lakes at low concentrations [27,28,29,30]. Warming is referred to as a favorable factor for the recent spread of invasive species to northern Europe [31]. However, it has not been fully disclosed how nutrient enrichment and competition with native species could affect their establishment in new areas.

The current paper addressed the potential risk of alien species invading temperate freshwaters. The effect of nutrients and temperature on the strains of native *Planktothrix agardhii*, *Aphanizomenon gracile* and alien to Europe *Chrysosporum bergii* and *Sphaerospermopsis aphanizomenoides* cyanobacteria and their competition are highlighted. The aim of the study was to disclose proliferation, cyanotoxins synthesis, and interspecies competition of dominant native and alien cyanobacteria in temperate lakes under increasing temperature and eutrophication conditions.

## 2. Results

### 2.1. Experiment I: Effect of Nutrients on Native and Alien Cyanobacteria

#### 2.1.1. Effect of Nutrients on the Growth Rate of Cyanobacteria Strains

**Figure 1:**
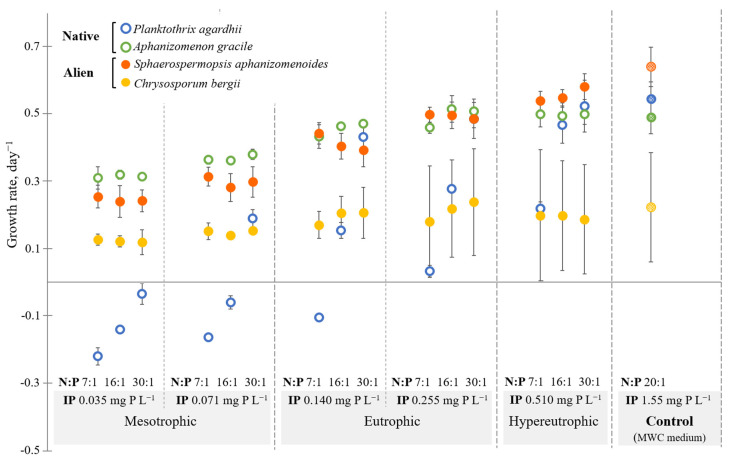
Growth rates (day^−1^) of the cyanobacteria species cultured under different nutrient concentrations and N:P ratio at 24 °C. Each symbol represents the average growth rate of two tested strains of each species at exponential growth phase. Data are reported as mean and error bars represent standard deviation.

The General linear model (GLM) showed that nutrient concentrations and N:P atomic ratio significantly affected the growth rate of all tested cyanobacteria strains (Table 1). However, the response was species- and strain-specific. The highest growth rates were found for strains of *Aphanizomenon gracile* (0.43 ± 0.08 day^−1^) and *Sphaerospermopsis aphanizomenoides* (0.40 ± 0.12 day^−1^) at all of the tested nutrient concentrations, and especially at more eutrophic and hypertrophic conditions (Figure 1). IP and IN impacted on both species growth rate, whereas for the *A. gracile* N:P ratio was also significant (Table 1). The lowest growth rate among the tested cyanobacteria was characteristic for *Planktothrix agardhii* strains (0.14 ± 0.25 day^−1^) and showed the biggest differences in growth under tested treatments. The maximum growth rate of *P. agardhii* was reached at the highest IP concentrations (0.51 mg P L^−1^) and at N:P ratio of 30:1, whereas low IP concentrations and N:P atomic ratio inhibited the cyanobacterium growth. The GML analysis revealed that the IP and IN concentrations, and N:P ratio were important for the growth of both strains of *P. agardhii*. The growth rate (0.18 ± 0.11 day^−1^) of *Chrysosporum bergii* was strain-specific and considerably differed under tested nutrient concentrations. The tested variables showed no significant impact on *C. bergii* growth rate (Table 1).

#### 2.1.2. Effect of Nutrients on Production of Cyanometabolites

**Figure 2:**
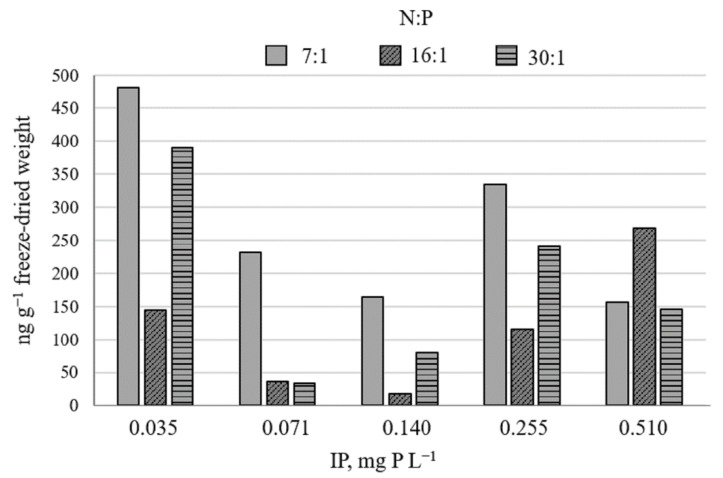
The amount of saxitoxin in *Aphanizomenon gracile* strain NRC_SIR/B41-09 grown under various IP concentrations and N:P ratio. Column represents cyanotoxin concentration in intermixed triplicate (*n* = 3) of each tested treatment.

The STX concentration in the experiment samples of the toxic *Aphanizomenon gracile* strain NRC_SIR/B41-09 ranged from 17.29 to 481.03 ng g^−1^ of freeze-dried weight (Figure 2). The highest toxin amount was detected at the lowest IP concentration (0.035 mg P L^−1^) and at N:P ratios 7:1 and 30:1. However, the regression analysis did not reveal a relationship between *A. gracile* growth rate, IP concentrations, N:P ratio to saxitoxin concentration (*p* > 0.05).

**Figure 3:**
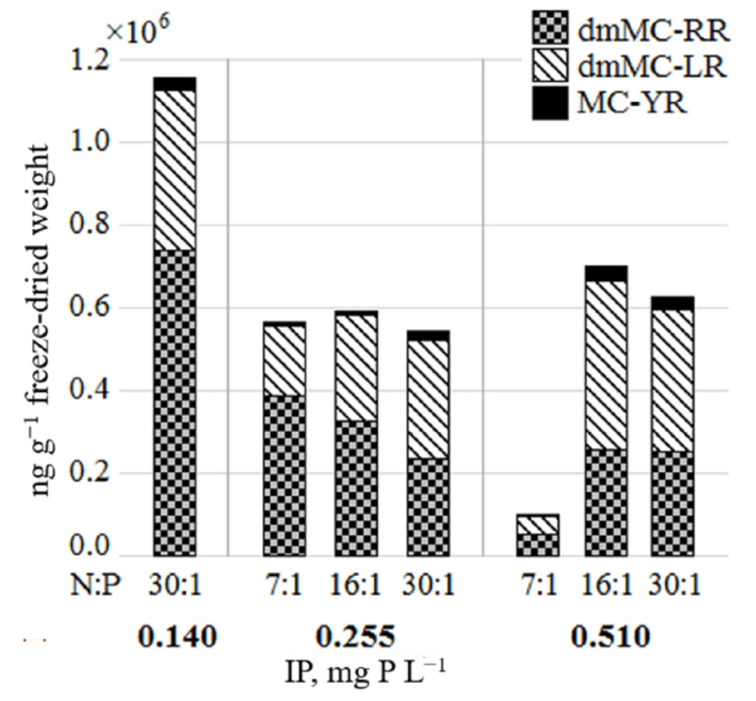
The amount of microcystins in toxic *Planktothrix agardhii* strain NRC_SIR/F5-09 under the tested IP concentrations and N:P ratio. Column represents cyanotoxins concentration in intermixed triplicate (*n* = 3) of each tested treatment.

Total MCs concentration in the toxic *Planktothrix agardhii* strain NRC_SIR/F5-09 ranged from 9.83 × 10^4^ to 1.16 × 10^6^ ng g^−1^ freeze-dried weight (Figure 3). The proportion of MCs variants slightly varied in all tested treatments. The demethylated microcystin RR (dmMC-RR) and demethylated microcystin LR (dmMC-LR) prevailed, and the highest content of the toxins was determined at the IP concentration 0.140 mg P L^−1^. The dmMC-RR content was up to 1.8-fold higher than dmMC-LR.

**Figure 4:**
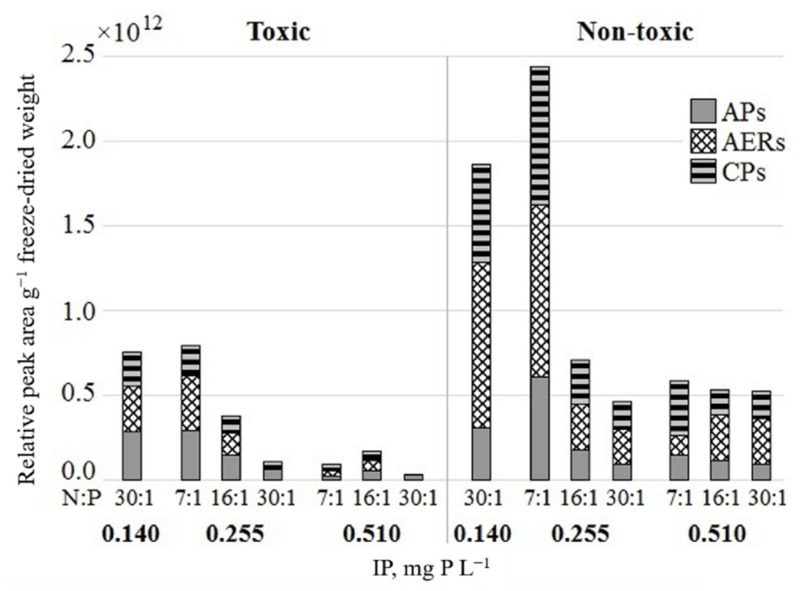
Relative number of oligopeptides in the biomass of toxic and nontoxic *Planktothrix agardhii* strains under the tested IP concentrations and N:P ratio. Classes of oligopeptides: APs—anabaenopeptins, AERs—aeruginosins, CPs—cyanopeptolins. Column represents oligopeptides amount in intermixed triplicate (*n* = 3) of each tested treatment.

Three oligopeptide classes, anabaenopeptins (APs), aeruginosins (AERs) and cyanopeptolins (CPs), were detected in *P. agardhii* toxic and nontoxic strains used in the experiment (Figure 4). APs and AERs were predominant and shared approximately an equal part of the total amount in the toxic strain. Contrary, AERs dominated over APs in the nontoxic strain. The relative total concentration of all oligopeptides in the nontoxic *P. agardhii* strain was up to 3-fold higher than in the toxic strain under all examined treatments.

The regression analysis revealed a strong negative relationship between IP and dmMC-RR, APs, CPs concentrations in toxic *P. agardhii* strain (*r* = −0.86, *r* = −0.81 and *r* = −0.85, *p* < 0.05, respectively), whereas dmMC-LR, MC-YR correlated positively (*r* = 0.78, *r* = 0.82, *p* < 0.05, respectively). The growth rate of nontoxic *P. agardhii* strain negatively correlated with relative amount of APs and CPs (*r* = −0.82, *r* = −0.78, *p* < 0.05, respectively).

The tested *P. agardhii* strains formed insufficient biomass at lower IP concentrations required for the analysis of secondary metabolites, and that prevented us from drawing clear conclusions on the nutrient effect on MCs and NRPs variation.

### 2.2. Experiment II: Interspecies Competition

The results of GLM analysis of biomass values on the final day of the experiment showed that nutrient (IP and IN) concentrations followed by temperature and species origin had the greatest effect on cyanobacteria species competition (Table 2). For native *Planktothrix agardhii* important factors were nutrients (IP and IN, *F*_(1, 16)_ = 37.33, *p* < 0.001) and alien species (*F*_(1, 16)_ = 9.05, *p* < 0.01), whereas for native *Aphanizomenon gracile* they were nutrients (*F*_(1, 16)_ = 16.44, *p* < 0.001) and temperature (*F*_(1, 16)_ = 6.86, *p* < 0.05). For alien *Sphaerospermopsis aphanizomenoides*, all the tested factors were significant (Table 2), whereas for *Chrysosporum bergii* only temperature was not a significant factor (*p* > 0.05).

**Figure 5:**
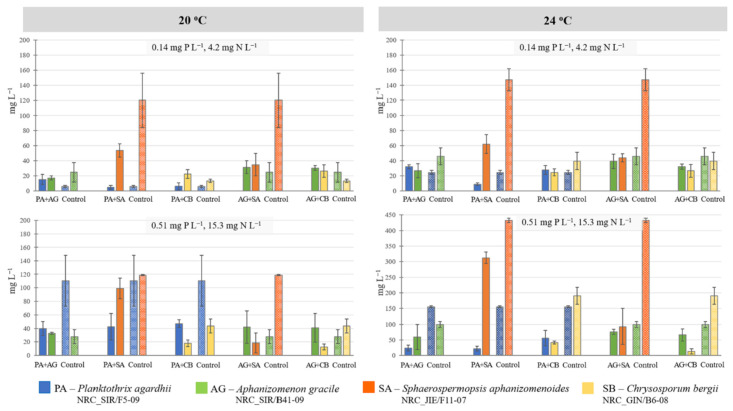
Biomass of co-cultured native toxins producing *Planktothrix agardhii*, *Aphanizomenon gracile,* and alien nontoxic *Sphaerospermopsis aphanizomenoides*, *Chrysosporum bergii* strains under different temperatures and IP concentrations (N:P ratio 30:1). Control strains were grown separately in the same medium and at the selected temperature. Data are reported as mean and error bars represent standard deviation.

Native *P. agardhii* and *A. gracile* competed for nutrients, especially in the treatments with higher IP and IN concentration (Figure 5). At the elevated nutrient concentrations and 24 °C temperature, the biomass of co-cultured *A. gracile* was 2.5 times higher (59.7 mg L^−1^) compared to *P. agardhii* (23.7 mg L^−1^). Compared to controls, *A. gracile* biomass in co-culture was approximately similar; however, the biomass of *P. agardhii* was 3–6.5 times lower in the treatments with higher IP and IN concentrations. This indicated that *P. agardhii* growth was suppressed by *A. gracile*.

Interactions among native and alien species were species-specific. The statistical analysis showed that alien species significantly affected growth of *P. agardhii* (*F*_(1, 16)_ = 9.05, *p* < 0.01); however, the effect of alien *S. aphanizomenoides* was more obvious compared to *C. bergii*. The biomass of co-cultured *P. agardhii* was 2.3–14 times lower compared to *S. aphanizomenoides*, especially in the treatment with elevated nutrient concentrations at 24 °C (21.8 mg L^−1^ vs. 312 mg L^−1^) (Figure 5). Co-cultured *P. agardhii* biomass was up to seven times lower compared to controls, while only a slight decrease of the biomass was seen for *S. aphanizomenoides*.

Availability of nutrients and temperature rather than interspecies competition had an effect on *P. agardhii* and *C. bergii* growth. *P. agardhii* biomass at elevated IP and IN concentrations was 2–7.4 times higher, whereas *C. bergii* built up the highest biomass in the treatments with high nutrients at 24 °C (Figure 5). Interspecies competition was more obvious in co-culture treatments at high nutrient concentrations and temperature, where species biomass was 2.8 and 4.6 times lower for *P. agardhii* and *C. bergii*, respectively.

*A. gracile* biomass in co-cultures was similar to *S. aphanizomenoides* and about two times higher than *C. bergii* (on average, 42.4 mg L^−1^ vs. 19.5 mg L^−1^) in most of the treatments. Higher nutrient concentrations predetermined a slight increase of *A. gracile* biomass and this was supported by GLM (*F*_(1, 16)_ = 16.44, *p* < 0.001 for IP and IN). At 20 °C temperature, *A. gracile* biomass in co-cultures with both alien cyanobacteria was 1.5 times higher than in the control treatments. Compared to the controls, *S. aphanizomenoides* biomass was 3.3–6.7 times lower, whereas *C. bergii* biomass was up to 15 times lower in co-cultures with native *A. gracile*. The assessment revealed that *A. gracile* did not suffer from alien species (Table 2), but had a negative effect on both alien species (*F*_(1, 16)_ = 61.18, *p* < 0.001 for *S. aphanizomenoides*; *F*_(1, 16)_ = 6.16, *p* < 0.05 for *C. bergii*).

## 3. Discussion

Temperature along with eutrophication stimulate an increase in frequency, duration, and intensity of harmful cyanobacterial blooms in freshwaters [9,32]. It is widely shown that global warming induces the expansion and introduction of alien species to Europe, altering the structure and functioning of native communities [33,34]. As cyanotoxins’ production in the water body is dependent on the contribution of toxigenic strains in cyanobacterial community, those changes might also lead to a shift in toxin composition and concentration. In the current study, the interplay of nutrients, temperature, and competition of native and alien cyanobacteria species growth, and cyanotoxin and/or other NRPs production were tested during complex controlled experiments with multiple factors and mixed species. The results discussed will give a better understanding about the complex role of abiotic and biotic variables, interspecies competition for the harmful cyanobacteria blooms, and species invasiveness to the temperate freshwaters under changing conditions.

### 3.1. Cyanobacteria Growth Response to Nutrients

The experimental studies revealed that nutrients (N:P ratio, IN, IP) were the key factors determining the growth rate of tested native and alien to Europe cyanobacteria. The response of cyanobacteria species to nutrients was species- and strain-specific. In addition, the environmental studies indicated that both phosphorus and nitrogen contributed and controlled the occurrence and intensity of the bloom [8,9,10,35,36], and the situation depends on the nutrient preferences of the potentially harmful cyanobacteria.

#### 3.1.1. Native Species Response

The growth rate of *P. agardhii* was mostly affected by the N:P ratio, followed by the changes in IP and IN (Table 1). Eutrophic and hypertrophic conditions with the N:P ratio 30:1 were the most favorable, whereas tested mesotrophic and eutrophic conditions with the N:P ratio 7:1 and 16:1 clearly limited growth of the species (Figure 1). These results agree with the data of field studies where common dominance and blooms of *P. agardhii* in hypertrophic lakes have been found [22,37,38]. *P. agardhii* suffers from nitrogen limitation at N concentrations lower than 0.05 mg L^−1^ [21], but is favored by high concentrations of phosphorus [39].

Similarly, IP followed by IN was the most significant factor for another native species *A. gracile* (Table 1). *A. gracile* growth rate increased gradually with increasing IP concentration. Figueiredo et al. [40] reported that the growth of the *Aphanizomenon flos-aquae* was highly dependent on phosphorus rather than on nitrogen, possibly due to the species N_2_ fixing capability. Figueiredo et al. [41] also showed experimentally that variation in nitrate level did not significantly affect the growth of *A. gracile*. In lakes, *A. gracile* thrive under nitrogen-deficient conditions [21] and at a high N:P ratio [15]. It suggests that *A. gracile* is highly adapted to various environmental conditions. The species is referred as a typical cyanobacterium for the temperate region and is characterized as a frequent dominant accompanied by other co-occurring prevailing species [21,30].

#### 3.1.2. Alien Species Response

Alien species responded differently to the varied nutrient concentrations. The experiment revealed that IP had a strong effect on the growth of the *S. aphanizomenoides* followed by IN. This is in agreement with Sabour et al. [42], who showed experimentally the maximum growth rate of *S. aphanizomenoides* under the highest nutrient concentrations; however, on the contrary, species reached an optimal growth rate at N:P ratios from 1 to 15. Figueiredo et al. [41] found that *S. aphanizomenoides* was moderately to extremely sensitive to nitrate depletion in the medium. This also supports the findings by Budzyńska and Gołdyn [43] from field studies as *S. aphanizomenoides* was characterized as a high-nutrient demanding species.

The current study disclosed that the growth potential of *C. bergii* was strain-specific, especially under hypertrophic conditions (Figure 1). Nutrient concentration and N:P ratio were not significant factors for *C. bergii* (Table 1). Other studies indicated that *C. bergii* preferred low concentrations of inorganic phosphorus and the species was the most powerful competitor among Nostocales at moderate (19–20 °C) temperatures [24,44]. On the contrary, Savadova et al. [29] revealed that the species has a preference for higher (26–30 °C) temperatures. This indicates that temperature rather than nutrients could be a limiting factor for the species proliferation in Lithuanian water bodies as it remains at low biomass in Lake Gineitiškės since 2008 (up to 0.26 mg L^−1^ [28]). In addition, it can explain the slow *C. bergii* spread northwards as only single filaments were found in Lake Rėkyva in 2014, the northernmost point of species distribution in Europe (unpublished data).

### 3.2. Cyanometabolites Production in Response to Nutrients

The stoichiometric theory of phytoplankton toxin regulation has stated that N limitation causes a reduction of N-rich toxins, whereas P shortage causes an increase in the most N-rich saxitoxins and the limitation of both nutrients promotes the C-rich toxins [45]. However, prediction of toxin type and concentrations in nutrient surplus conditions possibly does not follow those rules and is even more difficult to forecast.

Information on factors that regulate saxitoxin production in cyanobacteria is very limited. Kellmann et al. [46] analyzed the STX gene cluster in cyanobacteria and concluded that the target toxin synthesis may be regulated at the transcriptional level in response to the availability of phosphate and other environmental factors. The concentration of STX was notably higher in the biomass of *Raphidiopsis raciborskii* strain grown under oligotrophic rather than a super-eutrophic condition [47]. Nevertheless, though a similar tendency was observed in the current experiment, a significant relationship of *Aphanizomenon gracile* growth rate, IP concentrations, N:P ratio to STX concentrations was not revealed (*p* > 0.05).

A high variety of the MCs and bioactive non-ribosomal peptides was detected in scum samples of *P. agardhii* [48]. The current experimental study demonstrated that the toxic *P. agardhii* strain was able to produce three different variants of the MCs, and the shift in the proportion of MCs variants under varied nutrient conditions was not observed. Total concentration of the MCs was two times higher under eutrophic compared to hypertrophic conditions, but the total amount of toxins was not affected by the different N:P ratio. Therefore, these data cannot confirm the findings [16,49] that under N enrichment conditions higher MCs production is expected. Overall, complex environmental conditions (e.g., irradiance) rather than nutrients alone regulate mcy genes expression, and probably MCs production is mostly related to cell division and growth [50,51,52].

In the present study, tested *P. agardhii* strains produced APs, AERs and CPs, and the total amount of those NRPs was three-fold higher in nontoxic strain compared to MCs-producing strain. Our results are consistent with the data from other studies that highlighted co-production of various peptides with MCs in *P. agardhii* and the finding that strains lacking MC production contain other structurally related peptides that could play complementary [52].

Paerl et al. [53] concluded that toxin concentrations tend to be closely correlated with growth rate and cell abundance, and the factors that stimulate toxin biosynthesis may be group- or strain-specific. Nevertheless, the experiments revealed some regularities concerning the amount of the STX, MCs and other NRPs in relation to nutrient changes; however, further studies are needed to clarify the conclusions on this issue.

### 3.3. Combined Effect of Environmental Factors on Interspecies Competition

Competition is an important regulatory factor for community dynamics. The interspecies variation plays a crucial role in bloom dynamics; however, competition mechanisms between the different bloom-forming cyanobacteria species are poorly understood [41,54]. Eutrophication and climate warming simultaneously affect cyanobacteria community in natural ecosystems [32]. More complex and definitive suite experiments instead of the approaches focused on single stressors and individual species can more effectively capture the regularities of processes driven by eutrophication and a changing climate [55]. Laboratory culture and field experiments are the first steps towards understanding phytoplankton communities’ functioning and also can improve the confidence of predicting the success of alien species invasion.

The results of the current experiment revealed that IP together with IN concentrations had the greatest effect on cyanobacteria species competition, followed by temperature and species origin (Table 2). Native *A. gracile* suppressed growth of native *P. agardhii* and both alien species (Figure 5). Both native species evolved adaptations that help them to proliferate under nutrient-limiting conditions. *P. agardhii* can store nitrogen in a form of cyanophycin or phycocyanin [19] and surplus polyphosphate [56], whereas *A. gracile* can fix atmospheric N_2_ in heterocytes [57]. Teubner et al. [58] showed in field study that *Aphanizomenon flos-aquae* reached high biovolumes only when TN:TP < 16:1, while the growth of *P. agardhii* seems to be independent of seasonal variation of the TN:TP ratio. Our experiment did not reveal a significant dependence of the species competition on the N:P ratio. Possibly other factors or metabolites of *A. gracile* may significantly affect *P. agardhii* growth. It was found that an exudate of *A. gracile* had a cytotoxic effect on human neutrophiles [59] and the species extracts can induce cholinesterase activity in the fish brain homogenate [60]. More studies should be conducted to disclose the mechanisms of the interactions among the studied species.

The aim of the competition experiment was to assess the potential of alien cyanobacteria to outcompete native species that are dominant in eutrophic freshwaters of the temperate zone under ambient and elevated temperatures. The interspecies competition study under two different temperatures and IP concentrations revealed *Sphaerospermopsis aphanizomenoides* as a stronger invader compared to *Chrysosporum bergii*. Similarly, *S. aphanizomenoides* reached the highest growth rates and was the most powerful competitor followed by *Raphidiopsis raciborskii, Aphanizomenon gracile,* and *C. bergii* cyanobacteria in the experimental study performed by Mehnert et al. [44]. According to Savadova et al. [29], *S. aphanizomenoides* tolerated a wide range of temperatures (20–30 °C), and growth rate was one of the highest between tested native and alien cyanobacteria. These abilities probably predetermined increased proliferation and dominance of the species in temperate lakes [23,24,26]. The current experiment revealed that *S. aphanizomenoides* was a superior competitor over *P. agardhii* under elevated nutrient concentrations at 24 °C, but not in the case of *A. gracile,* which may suppress the establishment of alien cyanobacteria in temperate lakes. Several studies indicated the importance of secondary metabolites in the interspecies competition among cyanobacteria [54,61,62,63]. However, there is no evidence that alien species in Europe such as *Raphidiopsis raciborskii* can produce cyanotoxins [59]. Kokociński and Soininen [24] found a negative relationship between the native *A. gracile* biomass and the biomass of alien *Chrysosporum bergii* from field data analysis. Similarly, our experiment demonstrated a negative effect of STX producing *A. gracile* strain on other tested native *P. agardhii* and nontoxic alien cyanobacteria species isolated from the lakes in Lithuania.

## 4. Conclusions

Both native *Planktothrix agardhii*, *Aphanizomenon gracile,* and alien to Europe *Sphaerospermopsis aphanizomenoides* species successfully proliferated under elevated IP and IN conditions, whereas a high N:P ratio (30:1) was significant only for non-diazotrophic *P. agardhii*. The highest growth rate was detected for native *A. gracile* and alien *S. aphanizomenoides* under eutrophic-hypertrophic conditions, indicating the reason for their success in climate change conditions. The alien *Chrysosporum bergii* growth potential was not affected by nutrients.

A strain of native *A. gracile* species had the ability to produce STX, whereas *P. agardhii*—a complex of bioactive oligopeptides. The *P. agardhii* strain producing three variants of MCs (dmMC-RR, dmMC-LR, and MC-YR) had a 3-fold lower amount of other NRPs compared to MCs non-producing strain. The experiment showed that IP was important for the amount of MCs and/or other NRPs produced in different treatments. However, for both species further research is needed to determine the relationships between increase in nutrient concentration and toxin production.

The greatest effect on interspecies competition was under increased temperature coupled with elevated nutrient concentrations. Alien species of cyanobacteria differ by their invading abilities into native populations of temperate lakes. *S. aphanizomenoides* was a stronger invader compared to *C. bergii.* This study also showed that native cyanobacteria *A. gracile* can suppress establishment of alien species under warming and eutrophication conditions.

## 5. Materials and Methods

### 5.1. Experimental Approach

Two types of experiments were performed to assess nutrient effects on (i) cyanobacteria growth and toxic metabolites production and (ii) species competitive abilities, using eight strains belonging to potentially toxic native and nontoxic alien cyanobacteria that were isolated from Lithuanian lakes. Four of them were native strains: *Planktothrix agardhii* MCs producing and non-producing (further toxic and nontoxic strains, accordingly) and *Aphanizomenon gracile* STX producing and non-producing strains (further toxic and nontoxic strains, accordingly) (Table 3). Cyanobacteria species were identified and classified based on morphology after Komárek and Anagnostidis [64], Komárek [65]. The selected conditions in controlled growth chambers were ~ 90 µmol m^−2^ s^−1^ of light intensity and 16:8 light: dark day cycle. Illumination condition was chosen based on light preferences of native *P. agardhii* [66] and *A. gracile* [44] species. Cultures were grown in triplicate in Erlenmeyer flasks of 100 mL volume. The tested strains were re-isolated before the experiments to ensure low density of bacteria (<1%). Prior to the experiment, the strains were maintained for three days in MWC medium free of N and P elements at respective temperatures. An initial concentration of chlorophyll-*a* (chl-*a*) 10 ± 0.5 µg L^−1^ was used for all treatments, which reflected prebloom conditions according to WHO [2]. Flasks were manually mixed once a day during the 12-day experimental period.

### 5.2. Experiment I: Effect of the Nutrients

**Figure 6:**
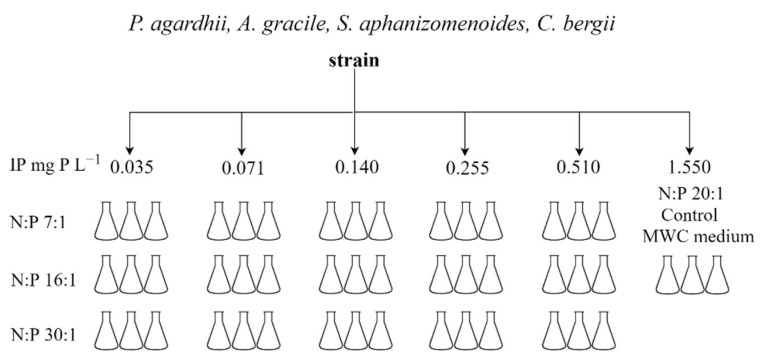
Schematic design of the experiment to test the effect of nutrients concentration on the growth rate and production of cyanotoxins and other NRPs of cyanobacteria strains.

The experiment was carried out to examine the effect of inorganic nitrogen (IN) and phosphorus (IP) concentrations, their atomic ratio (N:P) on the growth rate of cyanobacteria strains as well as on production of cyanotoxins and other non-ribosomal peptides (NRPs) (further cyanometabolites). Nitrogen and phosphorus free MWC medium was supplemented by phosphorus in the form of K_2_HPO_4_ at five different concentrations characteristic to temperate lakes of various trophy (based on Wetzel [67]): concentration 0.035 mg P L^−1^ corresponded to mesotrophic lakes, 0.071 and 0.140 mg P L^−1^ to eutrophic lakes, and 0.255 and 0.51 mg P L^−1^ to hypertrophic lakes (Figure 6). Nitrogen was added to the treatments in the form of NaNO_3_ according to N:P atomic ratio of 7:1; 16:1 and 30:1. Control treatments contained MWC medium (N:P atomic ratio 20:1, 1.55 mg P L^−1^). A temperature of 24 °C was chosen based on the results described in Savadova et al. [29]. The growth rate (µ) was evaluated by measuring chlorophyll-*a* content at the exponential growth phase of the strain using an AlgaeLabAnalyser (bbe Moldaenke GmbH, Schwentinental, Germany). At the end of the experiment, triplicate of each treatment of cyanometabolite producing strain were mixed in one sample, centrifuged at 8000× *g* for 6–12 min. supernatant was removed, and the biomass was freeze-dried. The obtained material was used to evaluate cyanotoxins and other NRPs.

#### 5.2.1. Evaluation of Growth Rate of Cyanobacteria Strains

The growth rate (chl-*a* day^−1^) was calculated according to Equation (1):

µ = ln (N_t_ − N_0_)/∆t (1)

where N_0_ and N_t_—chl-𝑎 values at the beginning and the end of the exponential growth phase, and Δt is the period of the exponential phase expressed in days [68].

#### 5.2.2. Analysis of Cyanometabolites

Freeze-dried cyanobacteria material was used for the analysis of intracellular amount of cyanometabolites. The extraction of microcystins (MCs) and other NRPs was performed using 75% methanol in MiliQ water. The saxitoxin (STX) was extracted with a mixture containing 4 mM ammonium formate buffer (pH 3.5) and acetonitrile (95:5, *v/v*) at a ratio of 2:3. All samples were disrupted by vortexing for 5 min and maintained for 5 min in a bath sonicator (Bandelin, Berlin, Germany), centrifuged at 10,000× *g* for 20 min, and the supernatant was transferred to chromatographic vials. The analysis was performed using liquid chromatography tandem with mass spectrometer LC-MS/MS (AB Sciex. Concorde, ON, Canada) equipped with a turbo ion spray ionization, operating in positive mode using the information-dependent acquisition method (IDA) for the detection of NRPs as described in Grabowska et al. [22]. The identification of NRPs (aeruginosins, anabaenopeptins, cyanopeptolins) was performed based on the enhanced ion product spectra. The relative amount of the NRPs was estimated and provided as the peak area in the extracted ion chromatogram. MCs quantitative analysis was performed by MRM (Multiple Reaction Monitoring) using standards for MC-LR, MC-RR, dmMC-LR, dmMC-RR, MC-YR, MC-LA, MC-LY, MC-LW, MC-LF variants (Alexis Biochemicals, San Diego, CA, USA). Detailed methodology presented in Khomutovska et al. [69]. Saxitoxin detection and quantitative analysis was conducted in MRM as described in Karosienė et al. [30] using STX standard (National Research Council, Halifax, Canada). Data were analyzed using Analyst QS^®^ 1.5.1 software.

### 5.3. Experiment II: Interspecies Competition

**Figure 7:**
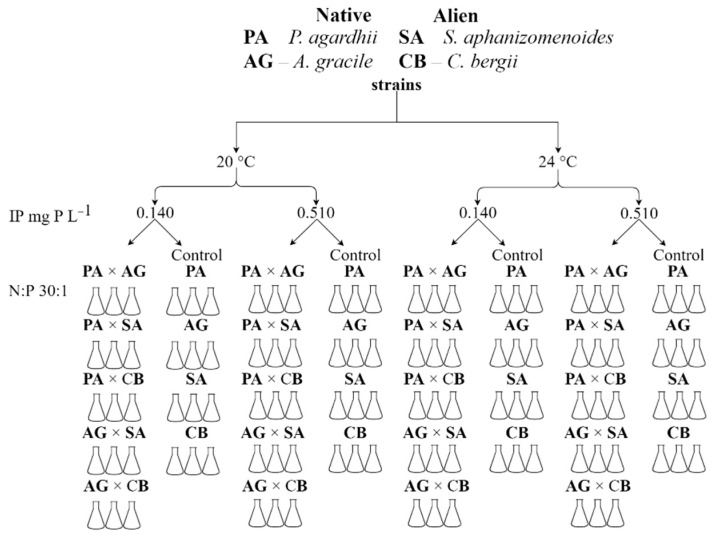
Schematic design of interspecies competition experiment.

Competitive properties of native *P. agardhii* and *A. gracile* cyanobacteria species and their ability to cope with alien *S. aphanizomenoides* and *C. bergii* under current (20 °C) climate conditions and in predicted warming (24 °C) and eutrophication scenarios (P 0.140; 0.51 mg P L^−1^ at N:P ratio of 30:1) were assessed. Toxic strains of native species of *P. agardhii* NRC_SIR/F5-09 (MCs producer) and *A. gracile* NRC_SIR/B41-09 (STX producer) were co-cultured together in one experimental setup with alien species *S. aphanizomenoides* NRC_JIE/F11-07 and *C. bergii* NRC_GIN/B6-08 strains. The control was each strain grown separately in the same medium and selected temperatures (Figure 7). The aliquot of 1 mL was removed from each treatment every fourth day and preserved with formaldehyde at the final concentration of 4%. The biomass changes were obtained by counting not less than 100 units (1 unit—100 µm of the filament) using Nageotte chamber with a light microscope [70]. Biomass was calculated based on the counted filaments number and mean filaments volumes using formulae for geometric shapes [71,72].

### 5.4. Statistical Analysis

General linear model (GLM) analysis was performed to analyze data of experiments and to reveal significant effects of the tested factors and their interactions on growth rate or biomass of the tested strains. The linear regression was used to assess the relationship between abiotic factors and cyanobacteria biomass, and the concentration of secondary metabolites. Before analysis all data were tested to satisfy normality assumption using Shapiro–Wilk tests. The transformation was not applied as the data were normally distributed. Statistical data analysis was processed using STATISTICA 6.0 software package (Stat Soft. Inc., Tulsa, OK, USA).

## Figures and Tables

**Table 1:** General linear model (GLM) results for factors’ impact on the response variable growth rate of cyanobacteria strains.

Response Variable (Growth Rate, day^−1^)	Factor			
	IP	IN	N:P	IP × N:P
Cyanobacteria	*F*_(32, 75)_ = 126.56 **	*F*_(112, 151)_ = 35.23 **	*F*_(16, 40)_ = 142.79 **	*F*_(64, 121)_ = 11.10 **
Species	*F*_(15, 168)_ = 54.31 **	*F*_(45, 9)_ = 35.94 *	*F*_(9, 270)_ = 554.91 **	*p* > 0.05
Strain	*F*_(35, 242)_ = 34.11 *	*F*_(91, 14)_ = 38.13 *	*F*_(21, 307)_ = 24.19 *	*p* > 0.05
Native/Alien	*F*_(5, 63)_ = 4.01 *	*F*_(15, 5)_ = 8.67 **	*F*_(3, 113)_ = 19.41 *	*p* > 0.05
*Planktothrix agardhii*	*F*_(4, 74)_ =660.16 *	*F*_(14, 74)_ =321.83 **	*F*_(2, 74)_ =800.00 *	*F*_(8, 74)_ = 28.06 *
*Aphanizomenon gracile*	*F*_(4, 75)_ = 183.78 *	*F*_(14, 75)_ = 54.44 **	*F*_(2, 75)_ = 6.03 *	*p* > 0.05
*Sphaerospermopsis aphanizomenoides*	*F*_(4, 73)_ = 226.29 *	*F*_(14, 73)_ = 66.14 **	*p* > 0.05	*p* > 0.05
*Chrysosporum bergii*	*p* > 0.05	*p* > 0.05	*p* > 0.05	*p* > 0.05

N:P, N and P atomic ratio; *, *p* < 0.001; **, *p* <0.01.

**Table 2:** General linear model (GLM) results for factors’ impact on the response variable biomass of cyanobacteria strains.

Response Variable (Biomass, mg L^−1^)	Factor					
	IP and IN	T	Species	Alien	Native	T × IP and IN
Cyanobacteria	*F*_(1, 112)_ = 29.50 *	*F*_(1, 112)_ = 23.10 *	*F*_(3, 112)_ = 14.02 *			*F*_(1, 112)_ = 15.44 *
*Planktothrix agardhii*	*F*_(1, 16)_ = 37.33 *	*p* > 0.05		*F*_(1, 16)_ = 9.05 **		*p* > 0.05
*Aphanizomenon gracile*	*F*_(1, 16)_ = 16.44 *	*F*_(1, 16)_ = 6.86 ***		*p* > 0.05		*F*_(1, 16)_ = 5.23 ***
*Sphaerospermopsis aphanizomenoides*	*F*_(1, 16)_ = 74.46 *	*F*_(1, 16)_ = 70.46 *			*F*_(1, 16)_ = 61.18 *	*F*_(1, 16)_ = 45.31 *
*Chrysosporum bergii*	*F*_(1, 16)_ = 7.11 ***	*p* > 0.05			*F*_(1, 16)_ = 6.16 ***	*F*_(1, 16)_ = 14.60 **

T, temperature; IP and IN, IP with N:P ratio of 30; *, *p* < 0.001; **, *p* <0.01; ***, *p* < 0.05.

**Table 3:** Cyanobacteria strains selected for the experiments.

Species		Strain	Lake	Cyanotoxins and NRPs	Other NRPs
Native	*Planktothrix agardhii*	NRC_SIR/F5-09	Širvys	MCs	NRPs
		NRC_JIE/E9-07	Jieznas	–	NRPs
	*Aphanizomenon gracile*	NRC_SIR/B41-09	Širvys	STX	–
		NRC_SIR/C10-07	Širvys	–	–
Alien	*Sphaerospermopsis aphanizomenoides*	NRC_JIE/G11-07	Jieznas	–	–
		NRC_JIE/F11-07	Jieznas	–	–
	*Chrysosporum bergii*	NRC_REK/D2-08	Rėkyva	–	–
		NRC_GIN/B6-08	Gineitiškės	–	–

MCs, microcystins; NRPs, non-ribosomal peptides; STX, saxitoxin; −, not detected.
